# Comparison of Salivary IGF-1, IGFBP-3, and CTX with Periodontal Status among Patients Belonging to Various Skeletal Maturity Groups

**DOI:** 10.3290/j.ohpd.b2805419

**Published:** 2022-03-14

**Authors:** Abdullah Almalki, Julie Toby Thomas, Mohamed Helmy Salama, Sara Ayid Alghamdi, Basim Almulhim, Abdullah Alassaf, Betsy Joseph, Ali Alqerban

**Affiliations:** a Assistant Professor, Department of Preventive Dental Sciences, College of Dentistry, Majmaah University, Al-Majmaah, Saudi Arabia. Study concept and design, funding acquisition, approved the final draft of the manuscript.; b Assistant Professor, Department of Preventive Dental Sciences , College of Dentistry, Majmaah University, Al-Majmaah, Saudi Arabia. Data acquisition, drafted the manuscript, approved the final draft of the manuscript.; c Assistant Professor, Department of Preventive Dental Sciences, College of Dentistry, Majmaah University, Al-Majmaah, Saudi Arabia; Department of Periodontics. College of Dentistry, Al-Azhar University, Cairo, Egypt. Revised the manuscript, approved the final draft of the manuscript.; d Assistant Professor, Department of Preventive Dental Sciences, College of Dentistry, Majmaah University, Al-Majmaah, Saudi Arabia. Reviewed and edited the manuscript, approved the final draft of the manuscript.; e Assistant Professor, Department of Preventive Dental Sciences, College of Dentistry, Majmaah University, Al-Majmaah, Saudi Arabia. Project administration, approved the final draft of the manuscript.; f Assistant Professor, Department of Preventive Dental Sciences, College of Dentistry, Majmaah University, Al-Majmaah, Saudi Arabia. Supervised the study, approved the final draft of the manuscript.; g Professor, Department of Periodontics, Saveetha Dental College and Hospitals, Saveetha Institute of Medical and Technical Sciences, Chennai, India. Data analysis and interpretation, approved the final draft of the manuscript.; h Associate Professor, Department of Preventive Dental Sciences, College of Dentistry, Prince Sattam Bin Abdulaziz University, Al-kharj, Saudi Arabia; Department of Preventive Dental Sciences, College of Dentistry, Dar al Uloom University, Riyadh, Saudi Arabia. Visualisation, approved the final draft of the manuscript.

**Keywords:** C-terminal telopeptide region of type I collagen, IGF-binding protein-3, insulin-like growth factors, periodontitis, skeletal maturity

## Abstract

**Purpose:**

To compare the levels of salivary IGF-1, IGFBP-3, and CTX with periodontal status among patients belonging to various skeletal maturity groups.

**Materials and Methods:**

This cross-sectional study was conducted on 80 participants 6 to 25 years of age. Based on skeletal maturity, the participants were categorised into 3 different stages: prepubertal, pubertal, and post-pubertal stages. The periodontal status of the participants was assessed using the simplified oral hygiene index (OHI-S), bleeding on probing (BOP), probing pocket depth (PPD), clinical attachment loss (CAL), and community periodontal index (CPI). The saliva samples were examined for IGF-1, IGFBP-3, and CTX using the respective ELISA kits. One-way ANOVA was used to determine statistically significant differences of means across the study groups for continuous variables.

**Results:**

The study demonstrated statistically significant differences for the parameters OHI-S, bleeding on probing, PPD, CPI, and CAL (p < 0.05) depending on skeletal maturity stage. ANOVA test showed a statistically significant difference by stage in IGF-1, IGFPB3, and CTX (p < 0.01).

**Conclusion:**

An association exists between periodontal status and levels of salivary IGF-1, IGFBP-3, and CTX in patients belonging to various skeletal maturity groups.

An interdisciplinary relationship between orthodontics and periodontal therapy facilitates achieving favourable outcomes in correction of malocclusion. The advent of prevention-based strategies has pointed out the relevance of assessing periodontal parameters, e.g. gingival bleeding, mobility, amount of keratinised tissue, gingival biotype, and presence of disease modifiers, before initiating orthodontic tooth movements. Orthodontic forces beyond the adaptive capacity of the periodontal ligament and alveolar housing during the phase of tooth activation and bone remodeling can lead to deleterious effects, such as rapid bone resorption (clinically manifested as gingival recession), bony dehiscence, fenestrations, and clinical attachment loss. Periodontal diagnosis before, during, and after active orthodontic treatment is well justified in terms of managing compromised cases involving pathological tooth migration, intra-bony defects, and trauma from occlusion.^13^

Lack of patient compliance with an oral-health–maintenance regime disrupts periodontal homeostasis due to a shift in microbial paradigm and progression of subgingival microbial dysbiosis. This manifests clinically as periodontal pockets, clinical attachment loss, gingival recession, bone loss, and tooth mobility, eventually leading to tooth exfoliation.^1,26^ Various other reasons contributing to microbial dysbiosis include physical, chemical and biological assault, which lead to periodontal inflammation, further favouring the release of specific nutritional and signaling molecules.^14^ An increased incidence of periodontitis among the younger age groups (8.6%) in the Saudi Arabian population compared to other developed countries emphasises the need to conduct awareness programs on preventive dental health care and on constraining detrimental habits such as smoking.^2^ Diet, stress, dental hygiene practices, alcohol consumption, and smoking are environmental factors that influence the composition and function of the oral microbiome.^24,39^ Supportive periodontal therapy includes preventive measures to control the dental biofilm, thereby controlling periodontal inflammation.

A high prevalence of clinical cases of malocclusion (88%) has been reported in those seeking orthodontic treatment, which is one of the predisposing factors for the progression of periodontal disease.^2^ Disruption of the qualitative monitoring functions of innate defense mechanisms and tissue maintenance by the commensal bacteria lead to excessive, uncontrolled inflammation and tissue destruction.^11^ Active orthodontic tooth movement can change the stress distribution on the periodontal ligament (PDL), resulting in disorientation and remodelling of the PDL fibers. Dysbiotic changes in the composition and number of bacteria in the oral microbiome can trigger a localised pro-inflammatory immune response at the oral barrier sites. The persistence of active periodontal disease enhances periodontal destruction by deregulating the process of bone remodeling; this occurs through disruption of the ‘coupling’ process between osteoclasts and osteoblasts.^20,32^ Hence, documentation of periodontal parameters in adjunct to radiographs as a routine orthodontic diagnostic procedure plays an imporant role in accomplishing successful orthodontic clinical outcomes.

Skeletal maturity is evaluated as a routine diagnostic step before initiating orthodontic therapy.^8,15^ This aids orthodontists in clinical decision-making related to the timing of faster bone acceleration and tooth movement. The evolution of molecular biology has prompted research on the role of biological growth mediators, e.g. onsulin-like growth factors (IGF-1), IGF-binding protein-3 (IGFBP-3), alkaline phosphatase (AP), and osteocalcin (OC), in predicting pubertal maturity and have been established as reliable indicators. These biomarkers for can also serve orthodontic diagnostic purposes to reduce unwanted additional radiation exposure during the orthodontic intervention.^9,33,40^

IGF-1, also known as somatomedin C, is a polypeptide hormone secreted mostly by the liver after the stimulation of growth hormone. IGF-1 is crucial in regulating postnatal longitudinal bone growth, and has also been reported to play a pivotal role in the acceleration and differentiation of substrate synthesis activity of osteoblasts and chondroblasts. Many studies^19,21,25,33^ have shown the importance of IGF-1/IGFBP-3 in tooth growth and development, as well as in the differentiation of human osteoblasts and human pulp fibroblasts. In addition to the IGF-dependent function of IGFBP-3 in transporting IGF-1 from its site of synthesis to its target cells, IGFBP-3 can interact with various extracellular proteins and proteins of plasma membrane, making its way into the cytoplasm and nucleus. Thus, IGFBP-3 plays a pivotal role in modulating various cellular functions, such as apoptosis.^33^

The dilemma in standardising reference values for these biomarkers arises due to other dependent factors, e.g. dysbiotic biofilm, genetic factors, and environmental factors, which promote pathogenesis and destruction of supporting tissues. Bone remodeling is controlled by the upregulation of circulating growth hormone (GH), IGFs, IGFBPs, and locally produced IGFs and IGFBPs, which have been demonstrated to elicit osteoblast differentiation by interacting with its respective receptors on the bone matrix.^33^

IGFs are bound to IGF-binding proteins, designated as IGFBP-1 to -6, and are expressed in biological fluids such as blood serum, cervical gingival fluid (GCF), or saliva.^21^ Toia et al^43^ stated that IGBF-3 is the most abundant IGFBP and regulates the amount of free, bioactive IGF-1, thus demonstrating its role in modulating bone cell proliferation. A study by Kanbur et al^25^ found a link between the maximum increase in serum IGF-1/IGFBP-3 molar ratio and the period of increased bone formation rate at the time of the pubertal growth spurt. This led them to conclude that the IGF-1/IGFBP-3 molar ratio may serve as a reliable method of determining the growth spurt.

A population-based study done in Pomerania, Germany, by Harb et al^26^ found that higher levels of periodontal disease were associated with low serum IGFBP-3 levels.^19^ Osteoclasts are considered the main culprits in inﬂammation-induced bone loss, as their increased numbers and hyperactivity are linked to active bone-resorption areas near the inﬂammatory inﬁltrate. In periodontal bone destruction, there are also regions with infection-induced inﬂammation which indicate a disturbance of tissue homeostasis and bone destruction. The presence of IGF-I and its receptor in periodontal tissues helps regulate the immune response by eliciting B-cell development, immunoglobulin formation, and interleukin-6 (IL-6) production during periodontal inflammation.^29^

Disturbed tissue homeostasis and alveolar bone destruction are mainly associated with an imbalance between osteoclastic and osteoblastic activity.^20^ Research on bone remodeling has targeted bone turnover biomarkers, such as AP, OC, C-terminal propeptide of type I procollagen (PICP), cross-linked C-terminal of type I collagen (ICTP), cross-linked C-terminal telopeptide of type I collagen (fragments alpha-CTX, beta-CTX) and N-terminal propeptide of type I procollagen (PINP). The C-terminal telopeptide region of type I collagen (CTX) is formed during bone degradation when insoluble collagen type 1 collagen is broken down in the resorption compartment of the osteoclast.^41^ Some researchers^7^ have demonstrated that CTX levels in oral fluids can serve as a potential diagnostic marker of periodontal disease, with high sensitivity and specificity for detecting increased bone destruction. As shown in the study by Betsy et al,^7^ the concentrations of salivary CTX, OC, and ON (osteonectin) could distinguish between subjects with periodontitis from those without.

In light of the current literature, it is necessary to emphasise the clinical relevance of periodontal diagnosis in orthodontic treatment planning and to examine whether non-invasive salivary biomarkers could help anticipate periodontal tissue destruction during orthodontic intervention. The prime objective of this study was to estimate the salivary levels of IGF-1, IGFBP-3, and CTX among the participants with and without periodontal disease belonging to the different skeletal maturity groups, categorised according to cervical vertebrae staging criteria. The study further examined the levels of these salivary biomarkers for possible association with periodontal disease severity.

## Materials and Methods

### Patient Selection

This cross-sectional study was conducted on a systemically healthy population (6-30 years old) between December 2020 and March 2021 at the College of Dentistry, Majmaah University, Zulfi, Saudi Arabia, after approval from the institutional ethics committee of Majmaah University, Saudi Arabia (Research Number: MUREC Nov.08/COM-2020/8-2; 8 November 2020) in compliance with the Helsinki Declaration. A convenience sample of 106 subjects who reported to the outpatient Department of Preventive Dental Science for orthodontic consultation participated in the study.

Exclusion criteria were any factors that can influence periodontal status, including:

- chronic systemic diseases
- diseases/factors affecting growth, such as vitamin D deficiency, parathyroid, growth and thyroid hormone disorders
- renal dysfunction
- diabetes mellitus
- growth abnormalities
- bleeding disorders
- need for bone metabolism medication in the previous six months
- xerostomia
- fixed or functional orthodontic treatment or radiotherapy
- pregnancy or lactation
- smokers

Before being enrolled, all study subjects who were 14 years old and parents of subjects under the age of 14 signed informed consent. Fourteen patients refused radiographic assessment, while 12 were uncooperative during saliva collection; thus, the total number of dropouts was 26. Finally, the complete data of 80 subjects were recorded and statistically analysed.

### Demographic Data and Radiographic Evaluation

Personal interviews were conducted with all study subjects, and data related to age, gender, place of origin, previous medical and dental history, family history of periodontal disease and diabetes, and personal oral hygiene habits were electronically recorded by the single examiner.

As a part of orthodontic diagnostic and treatment protocols, lateral cephalograms were taken for all recruited subjects for a duration of 1.25 s at 80 kVp, and 9 mA. Two examiners, blinded to the patient’s personal and clinical information, assessed the radiographs and broadly classified them into three groups based on skeletal maturity assessed by cervical vertebral staging (CS1-CS6) criteria:^3^

- Pre-pubertal (stage I) CS1: flat lower borders of C2, C3 and C4; the trapezoidal shape of the bodies of C3 and C4; CS2: concavity present at the lower border of C2; no change in the shape of bodies of C3 and C4.
- Pubertal (stage II) CS3: concavities at the lower borders of both C2 and C3; C3 and C4 bodies may be either be trapezoid or rectangular in shape; CS4: concavities at the lower borders of C2, C3, and C4; C3 and C4 bodies are rectangular horizontals in shape.
- Post-pubertal (stage III) CS5: concavities present at lower borders of C2, C3, and C4; Either one of the bodies of C3 or C4 is square in shape, others remain rectangular; CS6: concavities present at the lower borders of C2, C3, and C4; rectangular vertical shape of the bodies of C3 and C4.

### Clinical Parameters

Periodontal variables were examined and recorded using a mouth mirror and a community periodontal index (CPI) probe. For calibration, 15 participants were assessed by the primary investigator, and an intra-examiner calibration value of 0.86 was estimated using kappa statistics. Simplified oral hygiene index (OHI-S), bleeding on probing (BOP), probing pocket depth (PPD), clinical attachment loss (CAL), and community periodontal index (CPI) were measured to determine the periodontal status of all participants.^5,10,16^

The CPI scores assigned to the participants were based on the following criteria: CPI = 0, normal; CPI = 1, bleeding on probing and no pocket >3.5 mm; CPI = 2, calculus present and no pocket >3.5 mm; CPI = 3, shallow pocket 3.5–5.5 mm; CPI = 4, deep pocket ≥ 5.5 mm. The highest score was taken into consideration for the assessment of periodontal status.

The oral hygiene status of each participant was assessed using OHI-S. Labial and buccal surfaces of teeth,^11,16,26,31^ and the lingual surface of teeth 36 and 46 were used for assessment. The OHI-S score for each participant was derived by summing the debris index and calculus index scores. After recording individual tooth scores for debris and calculus, the debris index and calculus index were determined by adding the individual scores and dividing by the total number of teeth inspected. The results were categorised as excellent (0-1.2), fair (1.3-3), and poor (3.1-6).

CAL was measured as the distance from the cementoenamel junction to the bottom of the gingival sulcus. PD was measured from the gingival margin to the bottom of the sulcus. All measurements were taken at six tooth surfaces (mesiobuccal, mid-buccal, distobuccal, mesiolingual, mid-lingual, and distolingual). During the course of measurements, bleeding at any region was also recorded 10–15 s after probing. The presence of BOP was indicated with a plus sign (+).

### Salivary Sample Collection

To standardise diurnal variation, all individuals were instructed to report between the hours of 10 AM and 12 PM. They were instructed to sit upright in a comfortable position and thoroughly rinse their mouths before letting saliva accumulate in the mouth. The spitting method was used to collect 5 ml of unstimulated saliva in graduated Eppendorf tubes.^27^ The primary investigator labeled all the tubes, and the samples were stored at -20°C until further analysis. Biochemical analysis of saliva using enzyme-linked immune sorbent assay (ELISA) is described in detail in the supplement.

### Statistical Analysis

The continuous variables in the study, e.g. age and duration of appliance therapy, were summarised using mean ± SD. For categorical variables, frequency and percentage were used as summary measures. One-way ANOVA was applied to determine statistically significant differences of means across the study groups for continuous variables. Scheffe’s test was used for pairwise comparison of means of study groups All analyses were performed using SPSS version 20.0 (IBM; Armonk, NY, USA), and statistical significance was tested at a 5% level.

## Results

Out of the eighty participants, the number of participants classified based on skeletal maturity included 22 in the pre-pubertal group (stage I), 35 in the pubertal group (stage II), and 23 in the post-pubertal group (stage III). Mean ages in stages I, II, and III were 8.09 ± 1.97, 18.97 ± 4.11, and 22.74 ± 3.32, respectively.

Most patients in stages I and II were females, while in stage III, the majority were males. The majority of patients in all three stages lived in rural areas. Also, the majority of patients reported no family history of periodontal disease, and a toothbrushing frequency of once a day. The majority of patients in stage I visited the dentist once a year. The majority of patients in stage II and III visited the dentist at most twice a year (Table 1). Statistically significant differences between stages existed for the parameters OHI-S, bleeding on probing, PPD, CPI, and CAL (p < 0.05) (Table 2).

**Table 1:** Descriptive statistics of patients at various skeletal maturity by demographic variable

Variables		Stage I	Stage II	Stage III	Total
Gender	Male	7 (31.8%)	9 (25.7%)	14 (60.9%)	30 (37.5%)
	Female	15 (68.2%)	26 (74.3%)	9 (39.1%)	50 (62.5%)
Location	Urban	0	14 (40%)	11 (47.8%)	25 (31.2%)
	Rural	22 (100%)	21 (60%)	12 (52.2%)	55 (68.8%)
Family history of periodontal disease	Yes	1 (4.5%)	7 (20%)	7 (30.4%)	15 (18.8%)
	No	21 (95.5%)	28 (80%)	16 (69.6%)	65 (81.2%)
Frequency of brushing	Once	16 (72.7%)	17 (48.6%)	15 (65.3%)	48 (60%)
	Twice	5 (22.8%)	15 (42.9%)	7 (30.4%)	27 (33.8%)
	Never or occasionally	1 (4.5%)	3 (8.5%)	1 (4.3%)	5 (6.2%)
Previous dental visits	Once a year	15 (68.2%)	8 (22.9%)	8 (34.8%)	31 (38.8%)
	Twice a year	2 (9.1%)	11 (31.3%)	2 (8.7%)	15 (18.8%)
	Occasionally	5 (22.7%)	8 (22.9%)	9 (39.1%)	22 (27.4%)
	Never	0	8 (22.9%)	4 (17.4%)	12 (15%)
Age	Mean ± SD	8.09 ± 1.97	18.97 ± 4.11	22.74 ± 3.32	17.06 ± 6.69

**Table 2:** Comparison of various skeletal maturity stages based on periodontal parameters

Variable		Skeletal maturity stages				
		Stage I	Stage II	Stage III	Total	p-value
OHI-S	Good	20 (90.9%)	15 (42.9%)	10 (43.5%)	45 (56.3%)	< 0.01**
	Fair	2 (9.1%)	15 (42.9%)	11 (47.8%)	28 (35%)	
	Poor	0	5 (14.2%)	2 (8.7%)	7 (8.7%)	
Bleeding on probing	no BOP	18 (81.8%)	14 (40%)	8 (34.8%)	40 (50%)	< 0.05*
	<10%	2 (9.1%)	6 (17.1%)	2 (8.7%)	10 (12.5%)	
	10–30%	2 (9.1%)	7 (20%)	4 (17.4%)	13 (16.3%)	
	>30%	0	8 (22.9%)	9 (39.1%)	17 (21.2%)	
Probing pocket depth	Normal	22 (100%)	26 (74.3%)	15 (65.2%)	63 (78.8%)	< 0.05*
	4-5 mm	0	7 (20%)	8 (34.8%)	15 (18.8%)	
	>5 mm	0	2 (5.7%)	0	2 (2.4%)	
Community periodontal index (CPI)	0	18 (81.8%)	13 (37.1%)	8 (34.8%)	39 (48.8%)	< 0.05*
	1	3 (13.6%)	4 (11.4%)	5 (21.7%)	12 (15.4%)	
	2	1 (4.6%)	11 (31.2%)	6 (26.1%)	18 (22.5%)	
	3	0	5 (14.3%)	4 (17.5%)	9 (11.3%)	
	4	0	2 (5.7%)	0		
Clinical attachment loss (CAL)	No CAL	22 (100%)	30 (85.7%)	16 (69.6%)	68 (85.4%)	0.02*
	1–2 mm	0	2 (5.7%)	5 (21.7%)	7 (8.8%)	
	3–4 mm	0	1 (2.9%)	2 (8.7%)	3 (3.8%)	
	5 mm and more	0	2 (5.7%)	0		

*Statistically significant at 5% level (p < 0.05); ** statistically significant at 1% level (p < 0.01). If the cell count was less than 5, then cells were either merged or a continuity correction was made.

ANOVA showed statistically significant differences between stages for IGF-1, IGFPB3, and CTX (p < 0.01). However, Scheffe’s post-hoc test showed no statistically significant variations in IGF-1, IGFPB3 between stages II and III (p > 0.05). The lowest IGF-1 level was reported for stage I (0.99 ± 0.81), and it differed statistically significantly from stages II (1.80 ± 0.62) and III (1.70 ± 0.85). The lowest IGFBP3 level was reported for stage I (3.32 ± 0.56), and it differed statistically significantly from stages II (3.78 ± 0.63) and III (3.75 ± 0.47). The lowest CTX level was reported for stage I (1.41 ± 0.50), followed by stages II (2.49 ± 1.51) and III (4.50 ± 2.18) (Table 3). The ANOVA test showed a statistically significant difference (p=0.041) in concentration of IGF-1 levels in stage I based on severity of periodontal disease (Table 4).

**Table 3:** Comparison of skeletal maturity stages based on salivary biomarkers

Variable	Skeletal maturity stages			
	Stage I	Stage II	Stage III	p-value
IGF-1	0.99 ± 0.81^b^	1.80 ± 0.62^a^	1.70 ± 0.85^a^	<0.01**
IGFBP3	3.32 ± 0.56^b^	3.78 ± 0.63^a^	3.75 ± 0.47^a^	<0.01**
COLLAGENTELEPEPTIDASE CTX1	1.41 ± 0.50^a^	2.49 ± 1.51^b^	4.50 ± 2.18^c^	<0.01**

*Statistically significant at 5% level (p < 0.05); ** statistically significant at 1% level (p < 0.01). Means with different superscripts denote statistically significant pairwise differences (Scheffe’s test).

**Table 4:** Variations in salivary biomarkers based on periodontal disease severity

Variable	Skeletal maturity stages	Periodontal disease severity			
		CPI score			
		0	1–2	≥ 3	p-value
IGF-1	Stage I	0.82 ± 0.65	1.73 ± 1.17	–	0.041*
	Stage II	1.95 ± 0.51	1.67 ± 0.68	1.78 ± 0.71	0.506 NS
	Stage III	2.11 ± 0.72	1.52 ± 0.92	1.38 ± 0.77	0.242 NS
IGFBP3	Stage I	3.39 ± 0.55	2.99 ± 0.55	–	0.207 NS
	Stage II	3.54 ± 0.68	3.96 ± 0.60	3.78 ± 0.49	0.224 NS
	Stage III	3.96 ± 0.40	3.65 ± 0.42	3.59 ± 0.65	0.282 NS
Collagen Telepeptidase (CTX1) <	Stage I	1.44 ± 0.51	1.29 ± 0.53	–	0.621 NS
	Stage II	3.04 ± 0.94	2.32 ± 1.73	1.81 ± 1.72	0.195 NS
	Stage III	4.59 ± 1.50	4.74 ± 2.48	3.68 ± 2.80	0.721NS

*Statistically significant at 5% level (p < 0.05), NS: not statistically significant.

## Discussion

Orthodontic treatment planning is often jeopardised due to periodontal disease. Identification of periodontal tissue destruction during orthodontic intervention using non-invasive methods could be helpful in proper monitoring and decision making. To the best of our knowledge, this is the first study that compared salivary IGF-1, IGFBP-3, and CTX to the associated periodontal status among patients belonging to various skeletal maturity groups in a Saudi Arabian population.

### Comparison of Skeletal Maturity Stages Based on Salivary Biomarkers

Although there are no similar studies for comparison, several studies reported variations in periodontal status as age increases.^6,38^ In the present study, periodontal parameters such as OHI-S, bleeding on probing, PPD, CPI and CAL varied based on skeletal maturity stages. This study also showed that salivary levels of IGF-1, IGFPB3, and CTX increased from skeletal maturity stage I to stage II, and then decreased in stage III. This differs from the usually expected increase in stage III and should be interpreted cautiously. One of the reasons could be that the six stages of CVM were combined to form 3 stages in the present study. Another reason could be some unknown confounding factors that were present in the most mature skeletal age group.^30^

Furthermore, IGF-1 is known to increase in adolescence due to increased hormones, mainly initiated by sex steroid and growth hormone. This could be the reason for the increased levels in stage II, where the mean age was 18.97 ± 4.11 years. Our study reported an increase IGF-1 level from stage I to stage III. This is agrees with other studies, that timing of IGF-1 is strongly correlated with increased skeletal maturity.^9^ Kanbur et al^25^ also found an increase in serum levels of IGF-1 and IGFBP-3 ratios in more mature skeletal stages. IGF-1 is also known to affect bone remodelling in periodontal tissue due to an increase in the levels of local BMP levels, as reported in patients with acromegaly.^6^

IGF-1-binding-protein-3 (IGFBP-3) serves as a regulator of cellular activity.^30^ In this study, the lowest IGFBP3 level was reported for stage I, corresponding to the lowest skeletal maturity. IGFBP-3 is known to supress proliferation of cells by a mechanism that competitively binds to IGF-1. This also affects cell proliferation irrespective of IGF-1, which could also explain variations in IGF-1 levels among the patients. Moreover, IGFBP-3 is present at high concentrations and could affect bone formation.^17^ IGFBP-3 suppresses bone formation both in an IGF-1-dependent and IGF-1-independent manner. The IGFBP-3/IGF-1 axis controls remodelling of bone by both accelerating and inhibiting osteoblast differentiation.^17^

Increased levels of CTX have been found during bone resorption and degradation. It is formed when insoluble type I collagen is split into smaller fragments within the osteoclasts.^12^ In this study, CTX levels were 1.44 ± 0.51 for periodontally healthy individuals, while others reported a high level of 14.45 ± 3.63 in healthy individuals.^7^ Likewise, the highest level of CTX found in the present study (4.74 ± 2.48 ng/ml) is lower than that reported in other studies. This could be due to CTX being below the level of detection in most subjects.^4^

### Variations in Salivary Biomarkers Based on Periodontal Disease Severity

In this study, IGF-1 varied based on periodontal disease severity. It was lowest in the healthy group (CPI-0) and highest in the group with the highest CAL. These findings differ from those of other studies^34,35^ that reported the lowest levels of IGF-1 in the periodontitis group rather than in the healthy group. Furthermore, IGF-1 and CAL had a negative correlation among periodontally diseased patients, while healthy patients showed a positive correlation.^35^ Enhanced osteogenic differentiation and mineralisation has been documented in the presence of IGF-1, which suggests it has a significant role in periodontal bone remodelling.^28^

Similar variation was seen in terms of IGFBP-3 among various groups of periodontal disease severity. It showed an upward trend from skeletal maturity stage I to III and a downward trend from periodontitis patients to healthy participants (CPI-0 to 3). This is contradictory to the findings of other authors.^42^ A positive correlation was seen between IGFBP-2 levels and periodontal parameters, but not between IGFBP-3 and periodontal parameters. Higher levels of IGFBP-2 were found in GCF at disease sites.^42^ Smoking, one of the major risk factors of periodontal disease, did not seem to be a major confounder of the reported clinical associations between IGF-1, IGFBP-3, or IGF-1/IGFBP-3 ratios and specific disease entities when gender and age were matched.^36^

While some studies demonstrated a potential diagnostic ability of CTX to identify periodontal destruction, others did not.^18,34,41^ In contrast to the present study, a difference in the CTX levels of periodontitis patients was found by Betsy et al.^7^ Variations could be due to the different age range chosen in this study. Mishra et al^31^ also found higher salivary levels of CTX in periodontitis patients than in healthy participants. Furthermore, the levels of CTX were higher in periodontitis than healthy participants when the GCF was analysed.^37^ CTX showed an increasing trend. However, CTX was reported to have a weakly positive correlation with probing pocket depth and alveolar bone loss in a study that compared smokers and non-smokers with periodontitis.^23^ Several studies have been done in acromegalic patients, as they have increased GH and IGF-1 levels. The IGF-I levels were comparable in tests and controls, whereas IGFBP-3 levels were statistically significantly higher in acromegalic patients than in the control group. The severity of periodontal disease was also higher in acromegalic patients with higher IGF-1 levels.^19^

### Study Limitations

A relatively small sample size is the major limitation of this study. Although a larger sample size was planned, the second wave of COVID-19 hindered the sample collection for many reasons, such as fewer patients reporting for the study. A study with a larger sample size is envisaged to identify periodontal tissue destruction during orthodontic intervention using a non-invasive method of salivary biomarkers.

## Conclusion

An association was found between salivary IGF-1, IGFBP-3, and CTX and the periodontal status among patients belonging to various skeletal maturity groups. Periodontal parameters and salivary biomarkers varied based on skeletal maturity levels. Further multicentric longitudinal studies with a larger sample size are needed to identify a novel marker and therapeutic target among these biomarkers in periodontal tissue destruction.

## Supplement

### Biochemical analysis of saliva using enzyme-linked immune sorbent assay (ELISA)

The stored saliva was thawed and centrifuged (Kenley, London, UK, rotor radius 7 cm) at 3500 RPM for 10 min to remove proteins; the clear supernatant was collected to estimate insulin-like growth factor 1 (IGF1); insulin-like growth factor binding protein 3 (IGFBP3) and ELISA kit for cross-linked C-telopeptide of type I collagen (CTXI). ELISA kits were procured from Cloud-Clone Corp (Katy, TX, USA) for insulin-like growth factor 1 (IGF1) (Product No. SEA050Hu) for insulin-like growth factor binding protein 3 (IGFBP3) (Product No. SEA054Hu), and for crosslinked C-telopeptide of type I collagen(CTXI) (Product No. CEA665Hu), which were used with minor modifications of the protocol provided by the manufacturer. The given procedures and reagents are suitable for estimation of these constituents in serum, and because saliva contains minute quantities of these constituents, the standard solution was diluted to very low concentrations. Consequently, the quantity of saliva and reagents used were doubled. The standard IGF1 in the stock solution provided with the kit was 20 ng/ml, for IGFBP3 200 ng/ml and for CTXI 30 ng/ml. This was diluted serially with two-fold dilutions using the standard diluent solution (SDS).

Two hundred microliters of standard solution and saliva sample solution were pipetted into each of the wells of the precoated ELISA plate and incubated for 1 h at 37°C, the liquid removed from each well. Then 200 µl of “prepared detection reagent A” from the kit was added. This was mixed and incubated for 1 h at 37°C and later aspirated and washed three times. Later, 200 µl of “prepared detection reagent B” was added and incubated for 30 min at 37°C; subsequently, it was aspirated and washed 5 times. To this, 180 µl of substrate solution was added and incubated for 10–20 min at 37°C. Finally, 100 µl of stop solution was added, and the optical density (OD) was read immediately in an ELISA reader at 450 nm. Saliva collection was done before the assessment of clinical parameters on the same day. The clinicians who interpreted the radiograph and as well as the statistician were blinded to the diagnosis of the patients in all three groups. To make the calculation easier, the OD value of the standard (x-axis) was plotted against the log of the concentration of the standard (y-axis). The best-fit straight line was drawn through the standard points, as determined by regression analysis, using plotting software curve expert version 2.6.4. The concentration of the biochemical constituents in the saliva samples was determined from this curve. Detection range -IGF1: -0.156–10 ng/ml, IGFBP3: -1.56–100 ng/ml CTX I: -0.123–10 ng/ml. Sensitivity: IGF1: -0.067 ng/ml, IGFBP3: -0.61ng/Ml, CTX I: -0.05 ng/ml.
